# Challenges Associated with Investigating *Salmonella* Enteritidis with Low Genomic Diversity in New York State: The Impact of Adjusting Analytical Methods and Correlation with Epidemiological Data

**DOI:** 10.1089/fpd.2022.0068

**Published:** 2023-06-15

**Authors:** Deborah J. Baker, Amy Robbins, Jennifer Newman, Madhu Anand, William J. Wolfgang, Damaris V. Mendez-Vallellanes, Samantha E. Wirth, Lisa A. Mingle

**Affiliations:** ^1^ New York State Department of Health, Wadsworth Center, Albany, New York, USA.; ^2^ New York State Department of Health, Bureau of Communicable Disease Control, Albany, New York, USA.

**Keywords:** genomic epidemiology, *Salmonella* Enteritidis, whole-genome sequencing, PulseNet

## Abstract

Defining investigation-worthy genomic clusters among strains of *Salmonella* Enteritidis is challenging because of their highly clonal nature. We investigated a cluster identified by core genome multilocus sequence typing (cgMLST) consisting of 265 isolates with isolation dates spanning two and a half years. This cluster experienced chaining, growing to a range of 14 alleles. The volume of isolates and broad allele range of this cluster made it difficult to ascertain whether it represented a common-source outbreak. We explored laboratory-based methods to subdivide and refine this cluster. These methods included using cgMLST with a narrower allele range, whole genome multilocus sequence typing (wgMLST) and high-quality single-nucleotide polymorphism (hqSNP) analysis. At each analysis level, epidemiologists retroactively reviewed exposures, geography, and temporality for potential commonalities. Lowering the threshold to 0 alleles using cgMLST proved an effective method to refine this analysis, resulting in this large cluster being subdivided into 34 smaller clusters. Additional analysis by wgMLST and hqSNP provided enhanced cluster resolution, with the majority of clusters being further refined. These analysis methods combined with more stringent allele thresholds and layering of epidemiologic data proved useful in helping to subdivide this large cluster into actionable subclusters.

## Introduction

*S**almonella enterica* serovar Enteritidis is the most common serotype of *Salmonella*, comprising ∼25% of all *Salmonella* isolates in New York State (NYS) (data not including New York City). This serovar is highly clonal, which presents challenges for identifying clusters that are of epidemiological significance.

*Salmonella* Enteritidis emerged as a significant foodborne pathogen in the mid-1980s (Rodrigue et al., 1990). It is estimated that 82% of *Salmonella* Enteritidis infections can be attributed to eggs and poultry products (Gould et al., 2013). Other foods implicated in *Salmonella* Enteritidis outbreaks have been ground beef (2012), pine nuts (2011), and alfalfa sprouts (2011) (Deng et al., 2015). In addition, specific strains have been associated with international travel (O'Donnell et al., 2014).

PulseNet USA is the national foodborne disease surveillance system of the United States coordinated by the Centers for Disease Control and Prevention (CDC). Its members consist of state and local laboratories and federal food regulatory agencies. Standardized protocols are used by all participants to subtype foodborne pathogens such as *Escherichia coli*, *Listeria*, and *Salmonella*. Data are analyzed using BioNumerics (Applied Maths, Sint-Martens-Latem, Belgium) and uploaded into a national database, which CDC monitors for trends and emerging foodborne outbreaks (Tolar et al., 2019). The PulseNet network began in 1996 and has been instrumental in detecting dozens of foodborne outbreaks, which have led to multiple product recalls. It is estimated that this surveillance network prevents ∼270,000 cases of foodborne illness and saves the United States over $500 million in medical costs and loss of productivity each year (Ribot et al., 2019; Scharff et al., 2016).

The PulseNet system began subtyping *Salmonella* in 1988 using Pulsed-Field Gel Electrophoresis (PFGE) (Ribot et al., 2006; Swaminathan et al., 2001). In 2019, PulseNet transitioned from PFGE to Whole-Genome Sequencing (WGS) for foodborne outbreak surveillance with core genome multilocus sequence typing (cgMLST) and whole-genome multilocus sequence typing (wgMLST) being the analysis methods used in BioNumerics. These methods provide enhanced resolution when compared to PFGE (Kubota et al., 2019). In cgMLST analysis, a prescribed set of core genes are compared against a reference database for allelic differences, whereas with wgMLST, variable accessory genes are also included in the comparison (Besser et al., 2019; Jagadeesan et al., 2019).

With the transition to WGS, PFGE pattern assignments have been replaced with allele codes within PulseNet as a means of standardized nomenclature. An allele code, which consists of up to six digits, is assigned based on the relatedness of the isolate at specific allelic thresholds to other isolates in the PulseNet database The more the digits in common between isolates, the closer the genetic relationship (Nadon et al., 2017; Stevens et al., 2022; Tolar et al., 2019).

However, since widespread use of WGS for foodborne pathogens is still relatively new, the number of allelic differences which indicate a common source in an outbreak investigation is still uncertain. Establishment of these thresholds must be interpreted in conjunction with epidemiological information and will likely need to be adjusted based on outbreak characteristics or serovar (Besser et al., 2019; Rounds et al., 2020; Smith et al., 2020).

For many organisms, WGS provides a higher level of genetic discrimination and is demonstrably useful for aiding epidemiological investigations (Deng et al., 2021; Jagadeesan et al., 2019; Taylor et al., 2015). However, clonal pathogens such as *Salmonella* Enteritidis continue to present a challenge where the low genetic diversity leads to large uninformative clusters (Taylor et al., 2015). We investigated one such cluster of 265 isolates with collection dates extending over a 2.5-year period. Our initial cluster inclusion definition was that an isolate had to be within 5 cgMLST alleles and collected within 60 days of any other isolate in the cluster. Because new additions to the cluster could be within 5 alleles to one isolate but greater than 5 alleles to another, the cluster diversity increased to 14 alleles in a phenomenon referred to as “chaining.”

The number of isolates, lack of genetic diversity, and expanding allele range of this cluster impeded an effective epidemiological investigation. We utilized the tools of cgMLST, wgMLST, and high-quality single-nucleotide polymorphism (hqSNP) to address the following questions: Does lowering the cgMLST cluster threshold aid in subdividing this cluster and improve the epidemiological relevance? Would the additional analysis of these subclusters using wgMLST and hqSNP aid in additional resolution?

## Methods

### Sample acquisition

Samples were received through our routine PulseNet surveillance activities from clinical laboratories throughout NYS (excluding NYC). In NYS, clinical laboratories are required to submit all *Salmonella* isolates to the Wadsworth Center for confirmation and sequencing (NYS Communicable Disease Guidelines, 2020).

### DNA extraction

DNA was extracted from overnight cultures using either the QIAcube or the QIAcubeHT (Qiagen, German town MD). Samples were quantitated using Quant-iT™ dsDNA High-Sensitivity Assay Kit and the Fluoroskan™ Microplate Fluorometer in 96-well format (ThermoFisher Scientific, Waltham, MA).

### Library preparation, sequencing, QA check, and data deposition

Library preparation and sequencing were performed at the Wadsworth Center Advanced Genomic Technologies Cluster (AGTC). Library preparation followed standard protocols for Illumina Nextera XT or Illumina DNA Prep kits. Sequencing was performed using either an Illumina MiSeq with 2X250 chemistry and Version 2 kits, or an Illumina NextSeq with 2X150 chemistry and Version 2 kits. NextSeq reads were demultiplexed using the Illumina BCL2FASTQ script (Illumina, San Diego, CA).

Sequence quality met minimum quality thresholds for *Salmonella* (average *de novo* coverage ≥30 × , Q score ≥30 and core percent ≥85) established by PulseNet using BioNumerics version 7.6.3. Reads and metadata were uploaded to the PulseNet USA national database and the National Center for Biotechnology Information Sequence Read Archive (NCBI SRA) (BioProject PRJNA230403). Allele codes were assigned from the PulseNet national database.

### Cluster and phylogenetic analysis

Cluster analysis was performed on two different platforms: (1) BioNumerics 7.6.3 using PulseNet parameters and (2) GalaxyTrakr (https://galaxytrakr.org). BioNumerics was used to build trees and cluster samples using cgMLST and wgMLST schemes with various allele thresholds to call clusters (Tolar et al., 2019). The cgMLST and wgMLST schemes interrogate ∼3000 and 25,000 loci, respectively, which provide enhanced resolution over conventional MLST, which focuses on only 7 housekeeping genes. Initially, a cgMLST cluster of *Salmonella* Enteritidis was defined in NYS as 3 isolates within 5 alleles within 60 days. This cluster was then refined into cgMLST subclusters by lowering the allele threshold to 0 alleles within 60 days.

**FIG. 1.**
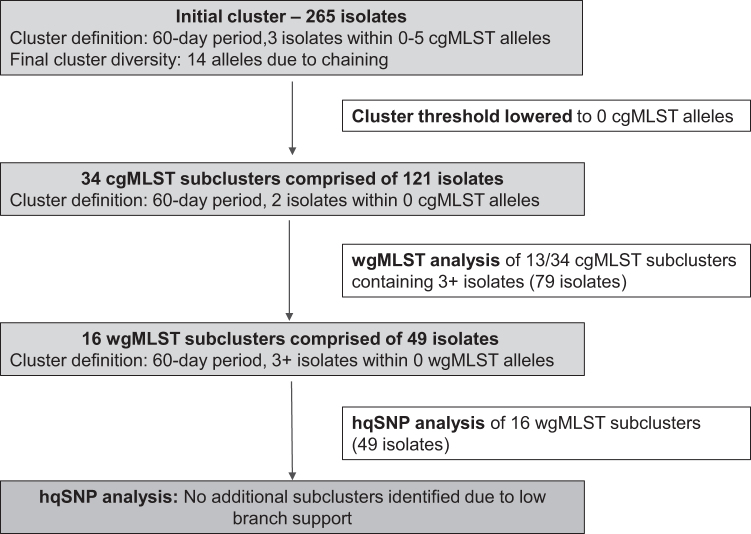
Analysis methods used to refine persisting *Salmonella* Enteritidis cluster in New York State. The initial cgMLST cluster of *Salmonella* Enteritidis was defined as 3 isolates within 5 alleles within 60 days. Retrospectively, this cluster was then refined into cgMLST subclusters by lowering the allele threshold to 0 alleles within 60 days. Further refinement of the 0 allele cgMLST subclusters containing 3 or more isolates was performed by wgMLST analysis of those subclusters with a threshold of 0 wgMLST alleles. The wgMLST subclusters were additionally assessed by hqSNP analysis. cgMLST, core genome multilocus sequence typing; hqSNP, high-quality single-nucleotide polymorphism; wgMLST, whole-genome multilocus sequence typing.

**FIG. 2.**
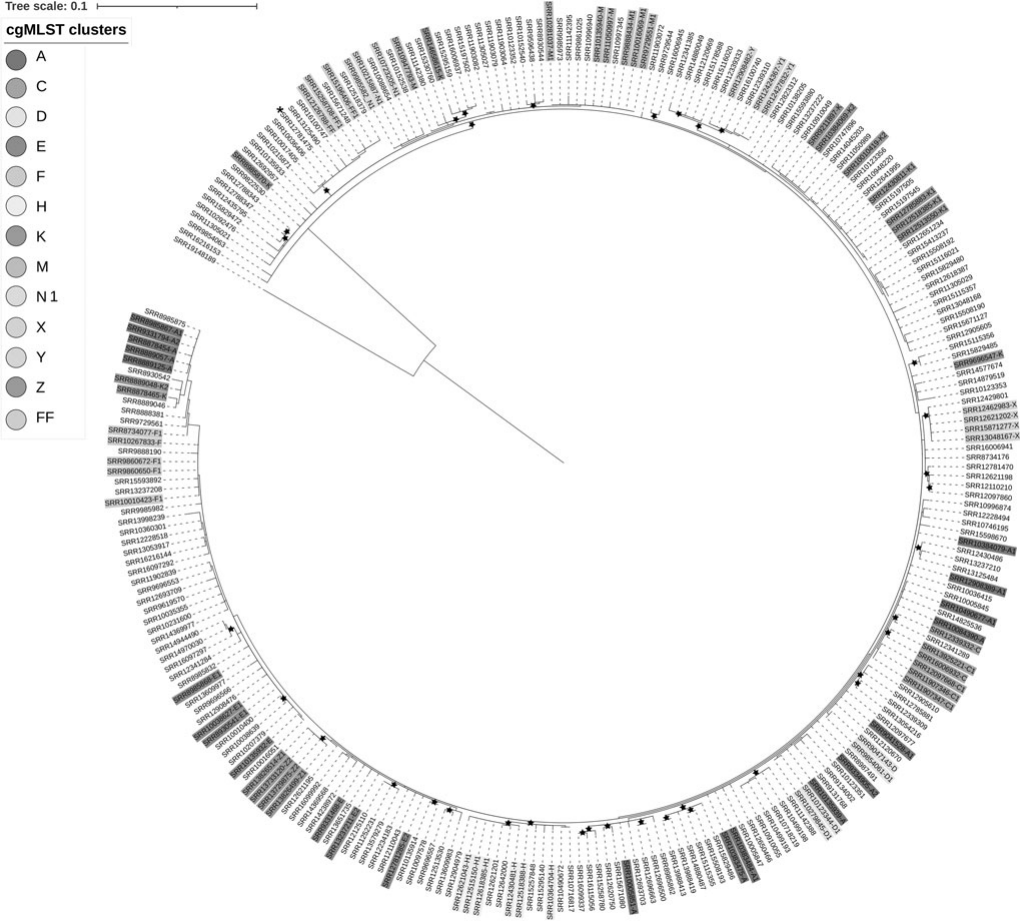
The maximum likelihood tree for the 265 SALM1.0 − 6743.2.4 × samples. The cgMLST- and wgMLST-clustered samples investigated are color coded and indicated by the text in the leaves. The tree is rooted at the midpoint and SRR19148189 is an outlier sample (not part of the SALM1.0 − 6743.2.4 × ). Excluding this outlier sample the entire SNP diversity of the tree is 33 SNPs. Branches with greater than 80% support are indicated by a star. Asterisk = reference genome.

Further analysis of the resulting subclusters was performed using wgMLST with a threshold of 0 alleles (Fig. 1). Analysis by hqSNP was performed with the FDA-Center for Food Safety and Applied Nutrition (CFSAN) SNP pipeline available in GalaxyTrakr using default settings (Davis et al., 2015). Reads were assembled for all isolates using Spades (GalaxyTrakr Version 3.11.1). To select a close reference to all samples (a requirement of the CFSAN pipeline), the SRR12781475 assembly was selected based on highest N50 (19 contigs with a total length of 4,691,766 bp) (QUAST GalaxyTrakr version 4.6.3). This reference was within 27 SNPs to the other 265 isolates run through the pipeline. Phylogenetic trees were built using RAxML (ver. 8.2.4 general time reversible maximum likelihood [ML] model) (Stamatakis, 2014) also available on the GalaxyTrakr. Branch reliability was tested with 500 bootstrap replicates (Fig. 2).

### Epidemiological investigation

Case investigation data for wgMLST subclusters consisting of more than two cases was retrospectively reviewed. Interview data were collected as part of routine surveillance. In subclusters where a specific food exposure was identified in more than 50% of cases, binomial probabilities were calculated. Statistical significance (*p* < 0.05) was calculated using the State of Oregon's Binomial Probability Worksheet (Oregon Health Authority Binomial Probability Worksheet) utilizing available background rates for NYS from the Centers for Disease Control and Prevention FoodNet 2018–2019 Population Survey (Centers for Disease Control). The original cluster was analyzed for food, water, animal, and travel exposure commonalities as well as by time and location using Statistical Analysis Software (SAS) version 9.4. This investigation is for public health activities and purposes, has been reviewed by the institutional IRB board and was determined to be exempt.

## Results

From January 2019 to August 2021, 781 *Salmonella* Enteritidis were received and analyzed. These 781 *Salmonella* Enteritidis were assigned to 82 unique allele codes when analyzed using PulseNet established parameters in the BioNumerics database. Of note, 81% (640/781 *Salmonella* Enteritidis) had the same allele code assignment of SALM1.0 – 6743.2.4 × . During this time frame, there were 22 named *Salmonella* Enteritidis clusters identified using cgMLST and an initial cluster definition of three isolates within five alleles in a 60-day period. Additional isolates were added to the clusters if they fell within 5 alleles and were within 60 days of any existing isolates. The 22 clusters contained 405 of the 781 *Salmonella* Enteritidis isolates. The largest cluster consisted of 265 isolates with allele code assignment of SALM1.0 – 6743.2.4 × (Supplementary Table S1). Due to chaining, the cgMLST allele range for this cluster expanded to 0–14 alleles.

Using both the cgMLST dendrogram and distance matrix (Supplementary Fig. S1), we identified 34 distinct subclusters where all pairwise distances were 0 alleles. Cluster sizes ranged from 2 to 15 isolates, with clusters harboring 121 (46%) of the 265 samples (Supplementary Table S1). All samples in these clusters had a neighbor outside of the cluster that differed by one or two alleles, highlighting how closely related all the isolates are to each other. Twenty-one of the subclusters comprised only two samples.

To improve resolution, we used a wgMLST scheme to examine each cgMLST subcluster individually. Analysis by wgMLST of each of the previously defined 0-allele cgMLST clusters resulted in pairwise wgMLST allele ranges of 0–1 to 0–7 (Table 1). Within each cgMLST subcluster, wgMLST analysis using a 0-allele cutoff was further discriminatory. Among the 13 cgMLST subclusters that had greater than two samples each, wgMLST analysis resulted in 16 wgMLST subclusters that had 0 allele pairwise diversity by both cgMLST and wgMLST (Table 1). Note: Cluster “X” did not have any isolates within 0 alleles by wgMLST and was not further investigated.

**Table 1.** Cluster Diversity and Epidemiological Links

Cluster (No. of isolates)	cgMLST (alleles)	wgMLST (alleles)	hqSNP diversity using internal reference	No. of cases interviewed	Epi links
***A* (15)**	0	0–4	0–11		
A1 (6)		0	0–4	5/6	5/5 Chicken
A2 (2)		0	0	2/2	2/2 Same grocery store, clustered in time, common food exposures (tangerines, cantaloupe, frozen vegetables)
***C* (6)**	0	0–2	0–6		
C1 (4)		0	0–4	¾	3/3 Chicken, beef, brand X peanut butter
***D* (4)**	0	0–3	0–2		
D1 (3)		0	0–2	3/3	3/3 Chicken
***E* (7)**	0	0–3	0–8		
E1 (3)		0	0	2/3	2/2 Chicken
E2 (2)		0	2	½	Insufficient epi data
***F* (5)**	0	0–1	0–8		
F1 (4)		0	0–8	4/4	3/4 Chicken, beef, grapes
***H* (6)**	0	0–3	0–6		
H1 (3)		0	0	3/3	3/3 Chicken, 2/3 same household
***K* (12)**	0	0–7	0–27		
K1 (4)		0	0–4	¼	Insufficient epi data
K2 (3)		0	2–13	1/3	Insufficient epi data
***M* (7)**	0	0–2	0–4		
M1 (4)		0	0	4/4	3/4 International travel to same country, 2/3 same resort
***N* (3)**	0	0	0–1		
N1 (3)		0	0–1	2/3	2/2 International travel to same country
***Y* (3)**	0	0–1	0–2		
Y1 (2)		0	0	½	Insufficient epi data
***Z* (4)**	0	0–1	0–1		
Z1 (2)		0	1	½	Insufficient epi data
Z2 (2)		0	0	2/2	2/2 Raw fish at same restaurant
**FF (3)**	0	0–1	1–3		
FF1 (2)		0	1	2/2	Household pair

Bold letters represent cgMLST defined clusters, letter/number combinations indicate wgMLST defined clusters.

cgMLST, core genome multilocus sequence typing; hqSNP, high-quality single-nucleotide polymorphism; wgMLST, whole-genome multilocus sequence typing.

Analysis using hqSNP increased the pairwise distance range for all the cgMLST clusters and some wgMLST clusters, although in most cases only slightly (Table 1). For all 265 samples, the overall SNP diversity was 33. In addition, the reliability of the branch positions in the maximum-likelihood tree was generally low (most below 80% bootstrap support), further reflecting the lack of genetic diversity among the samples (Fig. 2). For the clusters examined in Table 1, only isolates from cluster Z formed a well-supported (89% bootstrap) SNP cluster on the phylogenetic tree that aligned with their allele clusters (Fig. 2).

The most compelling epidemiological findings were among clusters designated as “A2,” “M1,” and “Z2.” Cluster A2 consisted of two cases with onsets of illness 4 days apart. These cases reported shopping at the same grocery store location that has a loyalty card system and had three food items reported in common (tangerines, cantaloupe, and frozen vegetables). Cluster M1 consisted of four cases, with three reporting travel to the same country during their incubation periods. Two of them traveled to the same resort. Cluster Z2 consisted of two isolates with onset of illness 2 days apart who reported consumption of raw seafood (*p* < 0.05) at the same restaurant location (Table 1).

Five of the 11 remaining subclusters had chicken identified as the food item in common, although this finding was not statistically significant (*p* > 0.05) for all subclusters reviewed. The only large cluster with an exposure that was statistically significant was cluster Z.

## Discussion

We explored ways to divide a large cluster with low genetic diversity and reviewed the epidemiology information on the resulting subclusters. With our previous cluster definition of three isolates within 5 cgMLST alleles in a 60-day period, a cluster of *Salmonella* Enteritidis with allele code SALM1.0 – 6743.2.4 × grew to 265 isolates over a period of two and a half years and did not identify statistically significant commonalities. The cluster was therefore not investigated after several months of analyzing by region, time period, links to live poultry, or international travel. Due to chaining, the cgMLST allele range of the cluster grew to 14. By lowering the cgMLST threshold to zero alleles, we subdivided this large cluster into 34 discrete, smaller subclusters. Thirteen of these 34 subclusters contained at least 3 isolates and were selected for investigation.

Further analysis of these 13 subclusters using wgMLST with a threshold of zero alleles revealed 16 additional subclusters of which only three had a strong epidemiological signal (clusters A2, M1 and Z2). Analysis by hqSNP increased resolution only slightly but because of low branch support in the ML tree, wgMLST and cgMLST clusters could not be reliably associated with SNP clusters, except for cluster Z. Thus, by subdividing the cluster using a reduced cgMLST allele cutoff of zero and then using wgMLST and hqSNP analyses, we were able to align genetic and epidemiological data in a small number of clusters. These were missed opportunities for public health action.

A limitation of this study was that the epidemiologic information was incomplete for cases in 31% of the subclusters and precluded examination of common exposures of those wgMLST clusters identified. NYS was heavily impacted by coronavirus disease 2019 (COVID-19) starting in March of 2020, with many public health personnel being diverted from foodborne investigations to assist in the COVID-19 response, which limited the ability to complete interviews. In addition, cases during this time often did not respond to interview requests.

## Conclusion

When we subdivided a large cluster of *Salmonella* Enteritidis using thresholds of 0 cgMLST and then 0 wgMLST alleles, small manageable clusters appeared, with several showing epidemiological connections. Our results are consistent with recent findings by Pan et al. (2022). The Pan et al. (2022) study concluded that cgMLST analysis of *Salmonella* offered excellent discriminatory power, but it was even greater when wgMLST was used, which is consistent with our results. Interestingly, the authors also found that CRISPR-MVLST (CRISPR multivirulence locus sequence typing) provided slightly better discriminatory power as cgMLST, nearly to the level of wgMLST. While we have not investigated CRISPR-MVLST in our laboratory as we routinely sequence all *Salmonella* isolates, it appears to be a comparable replacement to wgMLST for laboratories that are unable to routinely use WGS-based analysis.

Based on our investigation, we implemented a more stringent cluster threshold for *Salmonella* Enteritidis in January 2022, which specifies three isolates within 0 alleles by cgMLST in a 60-day time period. Additional analyses are needed to ascertain whether implementing this stricter threshold will aid in source identification, but initial impressions are that the efficiencies identified in investigating have been beneficial.

Our results show that when dealing with highly clonal strains such as SALM1.0 – 6743.2.4 × , epidemiological links are essential in identifying epidemiologically significant clusters. Laboratory data alone are not sufficient to pinpoint the discrete clusters within a large group of isolates that appear to be very closely related by cgMLST, wgMLST, and hqSNP analysis. However, when overlayed with robust epidemiologic investigations, hqSNP and wgMLST analysis can be useful additional tools to aid an investigation if an epidemiological link is discovered based on routine cgMLST analysis.

## Supplementary Material

Supplemental data

Supplemental data
