# Supplementary material for: Challenges Associated with Investigating Salmonella Enteritidis with Low Genomic Diversity in New York State: The Impact of Adjusting Analytical Methods and Correlation with Epidemiological Data
Source: Foodborne Pathog Dis. 2023 Jun 15;20(6):230–6. doi: 10.1089/fpd.2022.0068 (PMC10282972; doi:10.1089/fpd.2022.0068)
Supplement: Supplemental data [file Supp_TableS1.docx]

|  |  | **Table S1. Sample list for SALM1.0 - 6743.2.4x used in study** | | |  |  |  |
| --- | --- | --- | --- | --- | --- | --- | --- |
|  | cgMLST Cluster* | wgMLST Cluster** | Strain | BioSample Accesssion | SRA Accession | Isolation Date | Source |
| 1 | A |  | PNUSAS069217 | SAMN11392244 | SRR8878454 | 2/9/2019 | Human |
| 2 | A |  | PNUSAS069629 | SAMN11405573 | SRR8889057 | 2/13/2019 | Human |
| 3 | A |  | PNUSAS070074 | SAMN11405586 | SRR8889125 | 2/23/2019 | Human |
| 4 | A |  | PNUSAS091397 | SAMN12610392 | SRR10005851 | 7/17/2019 | Human |
| 5 | A |  | PNUSAS096582 | SAMN12714970 | SRR10084390 | 8/2/2019 | Human |
| 6 | A |  | PNUSAS099774 | SAMN12779905 | SRR10135939 | 8/15/2019 | Human |
| 7 | A |  | PNUSAS113450 | SAMN13176349 | SRR10383475 | 10/3/2019 | Human |
| 8 | A | A1 | PNUSAS072297 | SAMN11509856 | SRR8985867 | 3/24/2019 | Human |
| 9 | A | A1 | PNUSAS073477 | SAMN11619521 | SRR9041528 | 4/3/2019 | Human |
| 10 | A | A1 | PNUSAS113451 | SAMN13176348 | SRR10383484 | 10/3/2019 | Human |
| 11 | A | A1 | PNUSAS113463 | SAMN13176360 | SRR10384079 | 10/1/2019 | Human |
| 12 | A | A1 | PNUSAS117390 | SAMN13327807 | SRR10490677 | 10/18/2019 | Human |
| 13 | A | A1 | PNUSAS177540 | SAMN16564237 | SRR12908389 | 10/1/2020 | Human |
| 14 | A | A2 | PNUSAS077270 | SAMN12099312 | SRR9331794 | 5/15/2019 | Human |
| 15 | A | A2 | PNUSAS078989 | SAMN12107328 | SRR9334905 | 5/21/2019 | Human |
| 16 | B |  | PNUSAS159939 | SAMN15792530 | SRR12430486 | 7/10/2020 | Human |
| 17 | B |  | PNUSAS182257 | SAMN16881841 | SRR13125484 | 10/28/2020 | Human |
| 18 | C |  | PNUSAS156274 | SAMN15660539 | SRR12339332 | 7/1/2020 | Human |
| 19 | C |  | PNUSAS230056 | SAMN21552847 | SRR16006932 | 8/9/2021 | Human |
| 20 | C | C1 | PNUSAS146178 | SAMN15082754 | SRR11907346 | 3/22/2020 | Human |
| 21 | C | C1 | PNUSAS146180 | SAMN15082750 | SRR11907347 | 3/31/2020 | Human |
| 22 | C | C1 | PNUSAS149388 | SAMN15376517 | SRR12097668 | 5/7/2020 | Human |
| 23 | C | C1 | PNUSAS194447 | SAMN18242673 | SRR13925221 | 2/18/2021 | Human |
| 24 | D |  | PNUSAS073870 | SAMN11634388 | SRR9047143 | 4/12/2019 | Human |
| 25 | E |  | PNUSAS077269 | SAMN12098835 | SRR9331468 | 5/11/2019 | Human |
| 26 | E |  | PNUSAS099772 | SAMN12779907 | SRR10135932 | 8/17/2019 | Human |
| 27 | E | E1 | PNUSAS070782 | SAMN11461433 | SRR8930541 | 2/26/2019 | Human |
| 28 | E | E1 | PNUSAS072103 | SAMN11509852 | SRR8985868 | 3/20/2019 | Human |
| 29 | E | E1 | PNUSAS094662 | SAMN12661244 | SRR10038627 | 7/31/2019 | Human |
| 30 | E | E2 | PNUSAS172870 | SAMN16380647 | SRR12781285 | 9/3/2020 | Human |
| 31 | E | E2 | PNUSAS184323 | SAMN17050701 | SRR13237211 | 11/15/2020 | Human |
| 32 | F |  | PNUSAS108040 | SAMN13019899 | SRR10267833 | 8/22/2019 | Human |
| 33 | G |  | PNUSAS166103 | SAMN16093726 | SRR12621201 | 8/15/2020 | Human |
| 34 | G |  | PNUSAS167101 | SAMN16133897 | SRR12642000 | 8/18/2020 | Human |
| 35 | H |  | PNUSAS112663 | SAMN13158040 | SRR10364704 | 9/26/2019 | Human |
| 36 | H |  | PNUSAS159943 | SAMN15792526 | SRR12430481 | 7/21/2020 | Human |
| 37 | H |  | PNUSAS163307 | SAMN15905600 | SRR12518388 | 8/1/2020 | Human |
| 38 | I |  | PNUSAS123228 | SAMN13613656 | SRR10716817 | 10/19/2019 | Human |
| 39 | I |  | PNUSAS117397 | SAMN13327787 | SRR10490672 | 10/16/2019 | Human |
| 40 | J |  | PNUSAS137671 | SAMN14317190 | SRR11252231 | 2/12/2020 | Human |
| 41 | J |  | PNUSAS150701 | SAMN15415022 | SRR12125110 | 6/4/2020 | Human |
| 42 | K |  | PNUSAS069640 | SAMN11392240 | SRR8878465 | 2/4/2019 | Human |
| 43 | K |  | PNUSAS072669 | SAMN11509932 | SRR8985870 | 2/25/2019 | Human |
| 44 | K |  | PNUSAS077246 | SAMN11972721 | SRR9211897 | 5/8/2019 | Human |
| 45 | K |  | PNUSAS081695 | SAMN12288882 | SRR9696547 | 6/6/2019 | Human |
| 46 | K |  | PNUSAS203237 | SAMN19350670 | SRR14658615 | 5/2/2021 | Human |
| 47 | K | K1 | PNUSAS159980 | SAMN15792869 | SRR12430811 | 7/25/2020 | Human |
| 48 | K | K1 | PNUSAS162902 | SAMN15900670 | SRR12513550 | 8/3/2020 | Human |
| 49 | K | K1 | PNUSAS163304 | SAMN15905603 | SRR12518385 | 8/6/2020 | Human |
| 50 | K | K1 | PNUSAS173197 | SAMN16386626 | SRR12785883 | 9/13/2020 | Human |
| 51 | K | K2 | PNUSAS069219 | SAMN11405477 | SRR8889048 | 2/7/2019 | Human |
| 52 | K | K2 | PNUSAS091718 | SAMN12617016 | SRR10010419 | 7/17/2019 | Human |
| 53 | K | K2 | PNUSAS113462 | SAMN13176361 | SRR10384069 | 9/30/2019 | Human |
| 54 | L |  | PNUSAS138603 | SAMN14375423 | SRR11305027 | 2/19/2020 | Human |
| 55 | L |  | PNUSAS146053 | SAMN15077093 | SRR11903082 | 3/12/2020 | Human |
| 56 | M |  | PNUSAS099773 | SAMN12779906 | SRR10135940 | 8/13/2019 | Human |
| 57 | M |  | PNUSAS130246 | SAMN13898170 | SRR10947793 | 12/14/2019 | Human |
| 58 | M |  | PNUSAS133792 | SAMN14074893 | SRR11050997 | 1/12/2020 | Human |
| 59 | M | M1 | PNUSAS081715 | SAMN12435534 | SRR9888434 | 6/12/2019 | Human |
| 60 | M | M1 | PNUSAS081716 | SAMN12337483 | SRR9729551 | 6/12/2019 | Human |
| 61 | M | M1 | PNUSAS092230 | SAMN12627935 | SRR10016069 | 7/23/2019 | Human |
| 62 | M | M1 | PNUSAS108946 | SAMN13032440 | SRR10281037 | 9/12/2019 | Human |
| 63 | N |  | PNUSAS090125 | SAMN12587503 | SRR9985582 | 7/9/2019 | Human |
| 64 | N |  | PNUSAS104385 | SAMN12889247 | SRR10215887 | 9/5/2019 | Human |
| 65 | N |  | PNUSAS123594 | SAMN13615762 | SRR10723205 | 11/21/2019 | Human |
| 66 | O |  | PNUSAS079964 | SAMN12288880 | SRR9696553 | 5/31/2019 | Human |
| 67 | O |  | PNUSAS146010 | SAMN15076965 | SRR11902839 | 3/2/2020 | Human |
| 68 | P |  | PNUSAS079948 | SAMN12184531 | SRR9619570 | 5/29/2019 | Human |
| 69 | P |  | PNUSAS094248 | SAMN12657426 | SRR10035355 | 7/27/2019 | Human |
| 70 | Q |  | PNUSAS112573 | SAMN13153470 | SRR10360301 | 9/17/2019 | Human |
| 71 | Q |  | PNUSAS153315 | SAMN15543633 | SRR12228518 | 6/19/2020 | Human |
| 72 | R |  | PNUSAS091711 | SAMN12616994 | SRR10010400 | 7/15/2019 | Human |
| 73 | R |  | PNUSAS094527 | SAMN12661255 | SRR10038639 | 7/24/2019 | Human |
| 74 | S |  | PNUSAS169578 | SAMN16243411 | SRR12696500 | 8/31/2020 | Human |
| 75 | S |  | PNUSAS169660 | SAMN16244563 | SRR12696663 | 9/6/2020 | Human |
| 76 | T |  | PNUSAS149626 | SAMN15376926 | SRR12097860 | 5/8/2020 | Human |
| 77 | T |  | PNUSAS150111 | SAMN15400122 | SRR12110210 | 4/21/2020 | Human |
| 78 | U |  | PNUSAS117688 | SAMN13336327 | SRR10499198 | 10/20/2019 | Human |
| 79 | U |  | PNUSAS123304 | SAMN13613838 | SRR10718219 | 11/15/2019 | Human |
| 80 | V |  | PNUSAS092319 | SAMN12628340 | SRR10017405 | 7/24/2019 | Human |
| 81 | V |  | PNUSAS104377 | SAMN12889255 | SRR10215871 | 9/4/2019 | Human |
| 82 | W |  | PNUSAS173224 | SAMN16387707 | SRR12788343 | 9/14/2020 | Human |
| 83 | W |  | PNUSAS173225 | SAMN16387713 | SRR12788347 | 9/14/2020 | Human |
| 84 | X |  | PNUSAS161055 | SAMN15832914 | SRR12462983 | 7/27/2020 | Human |
| 85 | X |  | PNUSAS166106 | SAMN16093722 | SRR12621202 | 8/8/2020 | Human |
| 86 | X |  | PNUSAS180417 | SAMN16784709 | SRR13048167 | 10/12/2020 | Human |
| 87 | X |  | PNUSAS227217 | SAMN21409635 | SRR15871277 | 8/2/2021 | Food |
| 88 | Y |  | PNUSAS177549 | SAMN16564849 | SRR12908482 | 9/25/2020 | Human |
| 89 | AA |  | PNUSAS195143 | SAMN18332676 | SRR13988419 | 2/15/2021 | Human |
| 90 | AA |  | PNUSAS195144 | SAMN18332677 | SRR13988413 | 2/15/2021 | Human |
| 91 | BB |  | PNUSAS221263 | SAMN20846402 | SRR15508192 | 7/31/2021 | Human |
| 92 | BB |  | PNUSAS226352 | SAMN21367304 | SRR15829480 | 8/6/2021 | Human |
| 93 | CC |  | PNUSAS232066 | SAMN21882561 | SRR16099337 | 8/24/2021 | Human |
| 94 | CC |  | PNUSAS232324 | SAMN21895649 | SRR16115056 | 8/31/2021 | Human |
| 95 | D | D1 | PNUSAS083434 | SAMN12390569 | SRR9854061 | 6/25/2019 | Human |
| 96 | D | D1 | PNUSAS084660 | SAMN12758433 | SRR10123344 | 7/11/2019 | Human |
| 97 | D | D1 | PNUSAS108839 | SAMN13032162 | SRR10279845 | 9/12/2019 | Human |
| 98 | DD |  | PNUSAS209106 | SAMN19944123 | SRR14944490 | 6/3/2021 | Human |
| 99 | DD |  | PNUSAS209119 | SAMN19944154 | SRR14970030 | 6/9/2021 | Human |
| 100 | EE |  | PNUSAS207889 | SAMN19813183 | SRR14880049 | 6/2/2021 | Human |
| 101 | EE |  | PNUSAS211928 | SAMN20192742 | SRR15116020 | 6/23/2021 | Human |
| 102 | F | F1 | PNUSAS067599 | SAMN11149719 | SRR8734077 | 1/19/2019 | Human |
| 103 | F | F1 | PNUSAS083445 | SAMN12414033 | SRR9860650 | 6/27/2019 | Human |
| 104 | F | F1 | PNUSAS083450 | SAMN12414028 | SRR9860672 | 6/28/2019 | Human |
| 105 | F | F1 | PNUSAS091720 | SAMN12617028 | SRR10010423 | 7/13/2019 | Human |
| 106 | FF | FF1 | PNUSAS213833 | SAMN20322127 | SRR15196406 | 6/24/2021 | Human |
| 107 | FF | FF1 | PNUSAS215508 | SAMN20428515 | SRR15258708 | 6/30/2021 | Human |
| 108 | FF |  | PNUSAS150843 | SAMN15418194 | SRR12126788 | 6/11/2020 | Human |
| 109 | GG |  | PNUSAS211865 | SAMN20189118 | SRR15115355 | 6/17/2021 | Human |
| 110 | GG |  | PNUSAS221259 | SAMN20846405 | SRR15508193 | 7/7/2021 | Human |
| 111 | H | H1 | PNUSAS163032 | SAMN15901764 | SRR12515150 | 8/6/2020 | Human |
| 112 | H | H1 | PNUSAS165970 | SAMN16090146 | SRR12618385 | 8/9/2020 | Human |
| 113 | H | H1 | PNUSAS166095 | SAMN16093439 | SRR12621043 | 8/14/2020 | Human |
| 114 | HH |  | PNUSAS067346 | SAMN11149882 | SRR8734176 | 1/13/2019 | Human |
| 115 | HH |  | PNUSAS230054 | SAMN21552848 | SRR16006941 | 8/14/2021 | Human |
| 116 | Y | Y1 | PNUSAS159467 | SAMN15776556 | SRR12424367 | 7/8/2020 | Human |
| 117 | Y | Y1 | PNUSAS159714 | SAMN15784492 | SRR12427832 | 7/15/2020 | Human |
| 118 | Z | Z1 | PNUSAS193703 | SAMN18106851 | SRR13826499 | 1/26/2021 | Human |
| 119 | Z | Z1 | PNUSAS193706 | SAMN18106844 | SRR13826514 | 1/23/2021 | Human |
| 120 | Z | Z2 | PNUSAS192305 | SAMN17944879 | SRR13729875 | 1/22/2021 | Human |
| 121 | Z | Z2 | PNUSAS192319 | SAMN17964292 | SRR13733120 | 1/23/2021 | Human |
| 122 |  |  | PNUSAS092220 | SAMN12627909 | SRR10016051 | 7/16/2019 | Human |
| 123 |  |  | PNUSAS084622 | SAMN12412486 | SRR9861025 | 6/30/2019 | Human |
| 124 |  |  | PNUSAS090127 | SAMN12587512 | SRR9985973 | 7/9/2019 | Human |
| 125 |  |  | PNUSAS068138 | SAMN11404262 | SRR8888381 | 1/24/2019 | Human |
| 126 |  |  | PNUSAS069214 | SAMN11405382 | SRR8889046 | 2/4/2019 | Human |
| 127 |  |  | PNUSAS070087 | SAMN11457431 | SRR8930542 | 2/21/2019 | Human |
| 128 |  |  | PNUSAS070774 | SAMN11461442 | SRR8930544 | 3/1/2019 | Human |
| 129 |  |  | PNUSAS071471 | SAMN11509832 | SRR8985832 | 3/9/2019 | Human |
| 130 |  |  | PNUSAS072662 | SAMN11509937 | SRR8985875 | 3/26/2019 | Human |
| 131 |  |  | PNUSAS072663 | SAMN11509936 | SRR8985862 | 3/28/2019 | Human |
| 132 |  |  | PNUSAS073457 | SAMN11528499 | SRR8987491 | 3/29/2019 | Human |
| 133 |  |  | PNUSAS074339 | SAMN11871286 | SRR9131768 | 4/8/2019 | Human |
| 134 |  |  | PNUSAS074347 | SAMN11872605 | SRR9134002 | 4/8/2019 | Human |
| 135 |  |  | PNUSAS079001 | SAMN12139429 | SRR9596438 | 5/28/2019 | Human |
| 136 |  |  | PNUSAS079970 | SAMN12288889 | SRR9696557 | 6/7/2019 | Human |
| 137 |  |  | PNUSAS080766 | SAMN12288894 | SRR9696566 | 6/10/2019 | Human |
| 138 |  |  | PNUSAS081705 | SAMN12337411 | SRR9729544 | 6/14/2019 | Human |
| 139 |  |  | PNUSAS081724 | SAMN12337573 | SRR9729561 | 6/18/2019 | Human |
| 140 |  |  | PNUSAS082463 | SAMN12349172 | SRR9822530 | 6/24/2019 | Human |
| 141 |  |  | PNUSAS083427 | SAMN12390579 | SRR9854063 | 6/25/2019 | Human |
| 142 |  |  | PNUSAS084634 | SAMN12434500 | SRR9888190 | 7/2/2019 | Human |
| 143 |  |  | PNUSAS091393 | SAMN12610396 | SRR10005847 | 7/11/2019 | Human |
| 144 |  |  | PNUSAS091398 | SAMN12610391 | SRR10005845 | 7/18/2019 | Human |
| 145 |  |  | PNUSAS090134 | SAMN12587516 | SRR9985982 | 7/15/2019 | Human |
| 146 |  |  | PNUSAS098838 | SAMN12758921 | SRR10123351 | 7/29/2019 | Human |
| 147 |  |  | PNUSAS094516 | SAMN12658492 | SRR10036406 | 7/25/2019 | Human |
| 148 |  |  | PNUSAS098840 | SAMN12758931 | SRR10123352 | 7/29/2019 | Human |
| 149 |  |  | PNUSAS094524 | SAMN12658506 | SRR10036415 | 7/27/2019 | Human |
| 150 |  |  | PNUSAS098841 | SAMN12758930 | SRR10123353 | 7/30/2019 | Human |
| 151 |  |  | PNUSAS098842 | SAMN12758929 | SRR10123356 | 7/31/2019 | Human |
| 152 |  |  | PNUSAS096837 | SAMN12718026 | SRR10088654 | 8/10/2019 | Human |
| 153 |  |  | PNUSAS097774 | SAMN12726923 | SRR10097578 | 8/11/2019 | Human |
| 154 |  |  | PNUSAS099749 | SAMN12779903 | SRR10135914 | 8/8/2019 | Human |
| 155 |  |  | PNUSAS099768 | SAMN12779911 | SRR10135933 | 8/10/2019 | Human |
| 156 |  |  | PNUSAS100133 | SAMN12782272 | SRR10138205 | 8/14/2019 | Human |
| 157 |  |  | PNUSAS100750 | SAMN12796977 | SRR10152538 | 8/23/2019 | Human |
| 158 |  |  | PNUSAS100754 | SAMN12796970 | SRR10152540 | 8/18/2019 | Human |
| 159 |  |  | PNUSAS103675 | SAMN12876234 | SRR10207379 | 8/27/2019 | Human |
| 160 |  |  | PNUSAS105450 | SAMN12913055 | SRR10231600 | 9/5/2019 | Human |
| 161 |  |  | PNUSAS109090 | SAMN13040806 | SRR10292476 | 9/16/2019 | Human |
| 162 |  |  | PNUSAS117686 | SAMN13336329 | SRR10499193 | 10/21/2019 | Human |
| 163 |  |  | PNUSAS124198 | SAMN13656360 | SRR10746195 | 11/20/2019 | Human |
| 164 |  |  | PNUSAS124394 | SAMN13656852 | SRR10747896 | 11/25/2019 | Human |
| 165 |  |  | PNUSAS129863 | SAMN13878743 | SRR10910049 | 12/8/2019 | Human |
| 166 |  |  | PNUSAS129867 | SAMN13878885 | SRR10910055 | 12/12/2019 | Human |
| 167 |  |  | PNUSAS130248 | SAMN13898168 | SRR10948220 | 12/25/2019 | Human |
| 168 |  |  | PNUSAS132341 | SAMN13958400 | SRR10996874 | 1/9/2020 | Human |
| 169 |  |  | PNUSAS132345 | SAMN13958397 | SRR10996940 | 1/4/2020 | Human |
| 170 |  |  | PNUSAS132350 | SAMN13958409 | SRR10997345 | 1/8/2020 | Human |
| 171 |  |  | PNUSAS133782 | SAMN14074881 | SRR11050989 | 1/7/2020 | Human |
| 172 |  |  | PNUSAS135540 | SAMN14161577 | SRR11142380 | 1/27/2020 | Human |
| 173 |  |  | PNUSAS135541 | SAMN14161576 | SRR11142388 | 1/27/2020 | Human |
| 174 |  |  | PNUSAS135546 | SAMN14161596 | SRR11142395 | 2/2/2020 | Human |
| 175 |  |  | PNUSAS138588 | SAMN14375421 | SRR11305021 | 2/19/2020 | Human |
| 176 |  |  | PNUSAS138593 | SAMN14375416 | SRR11305029 | 2/10/2020 | Human |
| 177 |  |  | PNUSAS146040 | SAMN15077018 | SRR11903064 | 3/9/2020 | Human |
| 178 |  |  | PNUSAS146048 | SAMN15077069 | SRR11903072 | 3/7/2020 | Human |
| 179 |  |  | PNUSAS146055 | SAMN15077090 | SRR11903079 | 3/10/2020 | Human |
| 180 |  |  | PNUSAS149383 | SAMN15376530 | SRR12097677 | 4/29/2020 | Human |
| 181 |  |  | PNUSAS150117 | SAMN15400132 | SRR12110043 | 5/13/2020 | Human |
| 182 |  |  | PNUSAS150548 | SAMN15408049 | SRR12120670 | 5/20/2020 | Human |
| 183 |  |  | PNUSAS150549 | SAMN15408048 | SRR12120669 | 5/22/2020 | Human |
| 184 |  |  | PNUSAS153302 | SAMN15543612 | SRR12228494 | 6/15/2020 | Human |
| 185 |  |  | PNUSAS153524 | SAMN15548628 | SRR12234183 | 6/19/2020 | Human |
| 186 |  |  | PNUSAS156195 | SAMN15660403 | SRR12339309 | 6/20/2020 | Human |
| 187 |  |  | PNUSAS156196 | SAMN15660402 | SRR12339310 | 6/17/2020 | Human |
| 188 |  |  | PNUSAS156197 | SAMN15660544 | SRR12339333 | 6/25/2020 | Human |
| 189 |  |  | PNUSAS156269 | SAMN15663383 | SRR12341385 | 6/27/2020 | Human |
| 190 |  |  | PNUSAS156440 | SAMN15663160 | SRR12341289 | 7/5/2020 | Human |
| 191 |  |  | PNUSAS156441 | SAMN15663159 | SRR12341284 | 7/1/2020 | Human |
| 192 |  |  | PNUSAS159836 | SAMN15786781 | SRR12429801 | 7/16/2020 | Human |
| 193 |  |  | PNUSAS160108 | SAMN15800761 | SRR12435795 | 7/23/2020 | Human |
| 194 |  |  | PNUSAS162886 | SAMN15900648 | SRR12513530 | 8/3/2020 | Human |
| 195 |  |  | PNUSAS163302 | SAMN15905595 | SRR12518379 | 8/4/2020 | Human |
| 196 |  |  | PNUSAS165976 | SAMN16090140 | SRR12618387 | 7/27/2020 | Human |
| 197 |  |  | PNUSAS166065 | SAMN16093218 | SRR12620750 | 8/10/2020 | Human |
| 198 |  |  | PNUSAS166108 | SAMN16093720 | SRR12621198 | 8/10/2020 | Human |
| 199 |  |  | PNUSAS166110 | SAMN16093718 | SRR12621195 | 8/12/2020 | Human |
| 200 |  |  | PNUSAS167104 | SAMN16133894 | SRR12641995 | 8/16/2020 | Human |
| 201 |  |  | PNUSAS167477 | SAMN16179693 | SRR12650466 | 8/20/2020 | Human |
| 202 |  |  | PNUSAS169144 | SAMN16238698 | SRR12692957 | 8/27/2020 | Human |
| 203 |  |  | PNUSAS169276 | SAMN16239142 | SRR12693703 | 8/24/2020 | Human |
| 204 |  |  | PNUSAS167551 | SAMN16180014 | SRR12651234 | 8/21/2020 | Human |
| 205 |  |  | PNUSAS169282 | SAMN16239254 | SRR12693709 | 8/28/2020 | Human |
| 206 |  |  | PNUSAS172949 | SAMN16380758 | SRR12781475 | 9/4/2020 | Human |
| 207 |  |  | PNUSAS172955 | SAMN16380769 | SRR12781470 | 9/11/2020 | Human |
| 208 |  |  | PNUSAS173203 | SAMN16386631 | SRR12785881 | 9/8/2020 | Human |
| 209 |  |  | PNUSAS174719 | SAMN16435520 | SRR12823312 | 9/20/2020 | Human |
| 210 |  |  | PNUSAS177470 | SAMN16562241 | SRR12904979 | 9/19/2020 | Human |
| 211 |  |  | PNUSAS177480 | SAMN16562696 | SRR12905610 | 9/26/2020 | Human |
| 212 |  |  | PNUSAS177486 | SAMN16562700 | SRR12905605 | 9/29/2020 | Human |
| 213 |  |  | PNUSAS177550 | SAMN16564980 | SRR12908476 | 9/28/2020 | Human |
| 214 |  |  | PNUSAS180413 | SAMN16784692 | SRR13048168 | 10/11/2020 | Human |
| 215 |  |  | PNUSAS180541 | SAMN16790661 | SRR13053917 | 10/13/2020 | Human |
| 216 |  |  | PNUSAS180579 | SAMN16791543 | SRR13054216 | 10/21/2020 | Human |
| 217 |  |  | PNUSAS182262 | SAMN16881867 | SRR13125490 | 10/28/2020 | Human |
| 218 |  |  | PNUSAS184321 | SAMN17050668 | SRR13237210 | 11/17/2020 | Human |
| 219 |  |  | PNUSAS184327 | SAMN17050691 | SRR13237208 | 11/19/2020 | Human |
| 220 |  |  | PNUSAS184338 | SAMN17050759 | SRR13237222 | 10/20/2020 | Human |
| 221 |  |  | PNUSAS190839 | SAMN17692912 | SRR13579279 | 12/4/2020 | Human |
| 222 |  |  | PNUSAS191176 | SAMN17761173 | SRR13609983 | 12/20/2020 | Human |
| 223 |  |  | PNUSAS191180 | SAMN17761169 | SRR13609977 | 12/23/2020 | Human |
| 224 |  |  | PNUSAS195330 | SAMN18350783 | SRR13998239 | 2/19/2021 | Food |
| 225 |  |  | PNUSAS199893 | SAMN18935424 | SRR14369977 | 3/26/2021 | Food |
| 226 |  |  | PNUSAS213197 | SAMN20286094 | SRR15178588 | 6/4/2021 | Food |
| 227 |  |  | PNUSAS191696 | SAMN17834113 | SRR13651735 | 1/5/2021 | Human |
| 228 |  |  | PNUSAS195649 | SAMN18440218 | SRR14045203 | 3/1/2021 | Human |
| 229 |  |  | PNUSAS197873 | SAMN18742047 | SRR14238972 | 3/19/2021 | Human |
| 230 |  |  | PNUSAS199881 | SAMN18929731 | SRR14369568 | 3/29/2021 | Human |
| 231 |  |  | PNUSAS202063 | SAMN19241338 | SRR14577674 | 4/30/2021 | Human |
| 232 |  |  | PNUSAS206824 | SAMN19713097 | SRR14825536 | 5/26/2021 | Human |
| 233 |  |  | PNUSAS207872 | SAMN19813177 | SRR14880487 | 5/27/2021 | Human |
| 234 |  |  | PNUSAS207882 | SAMN19813191 | SRR14879519 | 5/21/2021 | Human |
| 235 |  |  | PNUSAS211856 | SAMN20189100 | SRR15115357 | 6/17/2021 | Human |
| 236 |  |  | PNUSAS211860 | SAMN20189106 | SRR15115356 | 6/17/2021 | Human |
| 237 |  |  | PNUSAS211927 | SAMN20192740 | SRR15116021 | 6/13/2021 | Human |
| 238 |  |  | PNUSAS216119 | SAMN20477950 | SRR15295159 | 6/21/2021 | Human |
| 239 |  |  | PNUSAS213835 | SAMN20325809 | SRR15197505 | 6/11/2021 | Human |
| 240 |  |  | PNUSAS213839 | SAMN20325821 | SRR15197502 | 6/15/2021 | Human |
| 241 |  |  | PNUSAS213840 | SAMN20325849 | SRR15197545 | 6/26/2021 | Human |
| 242 |  |  | PNUSAS215493 | SAMN20427071 | SRR15257848 | 7/1/2021 | Human |
| 243 |  |  | PNUSAS215597 | SAMN20429112 | SRR15258780 | 7/7/2021 | Human |
| 244 |  |  | PNUSAS216126 | SAMN20477943 | SRR15295140 | 7/9/2021 | Human |
| 245 |  |  | PNUSAS217542 | SAMN20525757 | SRR15330760 | 7/14/2021 | Human |
| 246 |  |  | PNUSAS219634 | SAMN20704198 | SRR15413237 | 7/12/2021 | Human |
| 247 |  |  | PNUSAS221257 | SAMN20846406 | SRR15508190 | 7/21/2021 | Human |
| 248 |  |  | PNUSAS222699 | SAMN20958655 | SRR15593880 | 8/1/2021 | Human |
| 249 |  |  | PNUSAS222704 | SAMN20958650 | SRR15593892 | 7/27/2021 | Human |
| 250 |  |  | PNUSAS222841 | SAMN20961031 | SRR15598670 | 8/4/2021 | Human |
| 251 |  |  | PNUSAS224241 | SAMN21040755 | SRR15671127 | 8/10/2021 | Human |
| 252 |  |  | PNUSAS224245 | SAMN21040781 | SRR15671248 | 8/12/2021 | Human |
| 253 |  |  | PNUSAS224248 | SAMN21040773 | SRR15671080 | 8/11/2021 | Human |
| 254 |  |  | PNUSAS226348 | SAMN21367308 | SRR15829486 | 8/16/2021 | Human |
| 255 |  |  | PNUSAS226351 | SAMN21367305 | SRR15829485 | 8/13/2021 | Human |
| 256 |  |  | PNUSAS226354 | SAMN21367302 | SRR15829472 | 8/12/2021 | Human |
| 257 |  |  | PNUSAS230048 | SAMN21552854 | SRR16006945 | 8/16/2021 | Human |
| 258 |  |  | PNUSAS231977 | SAMN21879971 | SRR16097297 | 8/23/2021 | Human |
| 259 |  |  | PNUSAS231980 | SAMN21879968 | SRR16097292 | 8/24/2021 | Human |
| 260 |  |  | PNUSAS230064 | SAMN21552863 | SRR16006937 | 8/22/2021 | Human |
| 261 |  |  | PNUSAS232067 | SAMN21882560 | SRR16099992 | 8/29/2021 | Human |
| 262 |  |  | PNUSAS232099 | SAMN21882935 | SRR16100747 | 8/16/2021 | Human |
| 263 |  |  | PNUSAS232104 | SAMN21882930 | SRR16100740 | 8/30/2021 | Human |
| 264 |  |  | PNUSAS234193 | SAMN22081199 | SRR16216153 | 8/26/2021 | Human |
| 265 |  |  | PNUSAS234199 | SAMN22081193 | SRR16216144 | 8/23/2021 | Human |
|  |  |  |  |  |  |  |  |
|  | *cgMLST cluster diversity 0 alleles  ** wgMLST cluster threshold 0-2 alleles | | |  |  |  |  |
