# Supplementary material for: Challenges Associated with Investigating Salmonella Enteritidis with Low Genomic Diversity in New York State: The Impact of Adjusting Analytical Methods and Correlation with Epidemiological Data
Source: Foodborne Pathog Dis. 2023 Jun 15;20(6):230–6. doi: 10.1089/fpd.2022.0068 (PMC10282972; doi:10.1089/fpd.2022.0068)

**Supplemental Figure 1**: The cgMLST dendrogram was built using BioNumerics 7.6.3 using PulseNet defined parameters. The 265 SALM1.0 - 6743.2.4x samples are shown in the dendrogram. When thresholds were set at 0 alleles for cluster definition 34 distinct subclusters were defined where all pairwise distances were 0 alleles. The cgMLST and wgMLST clustered samples investigated are color coded and indicated by the text in the nodes.


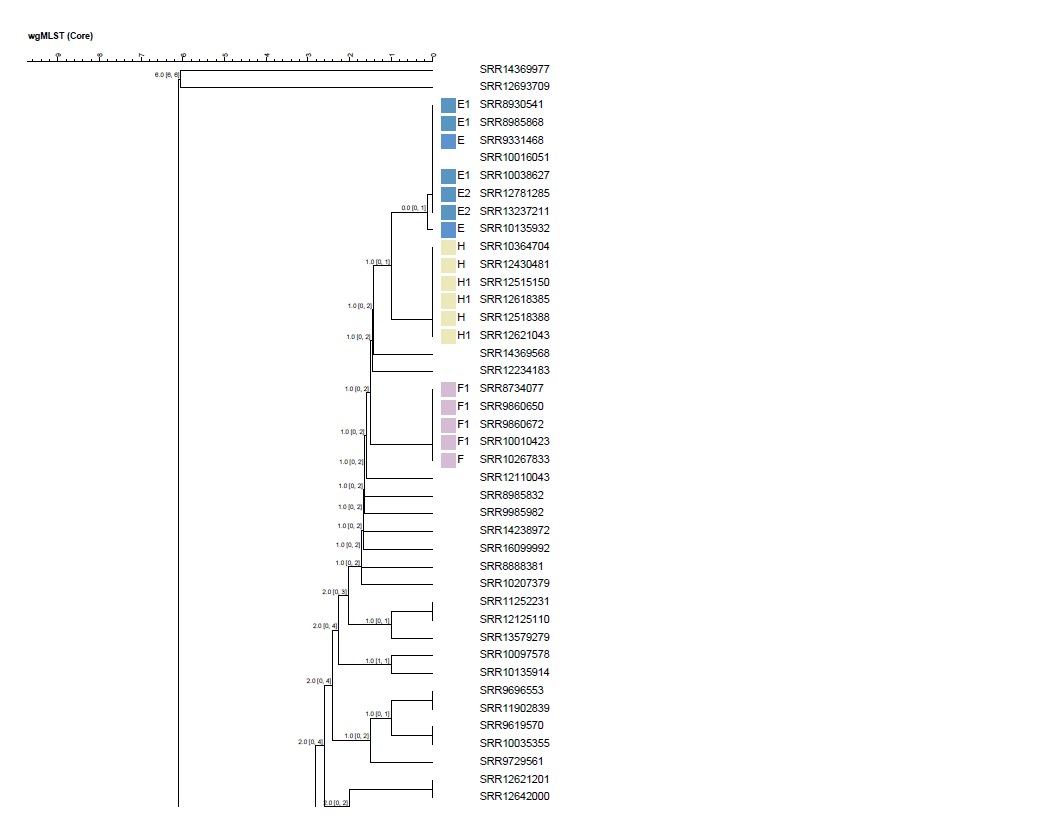


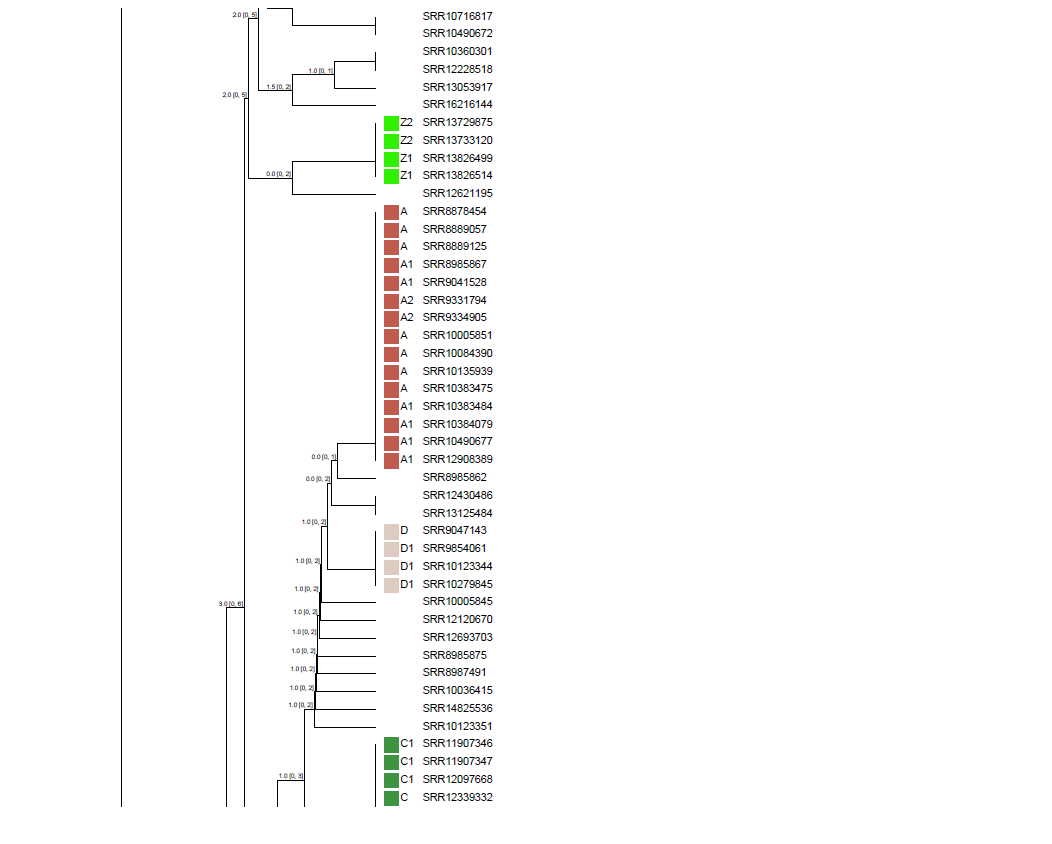


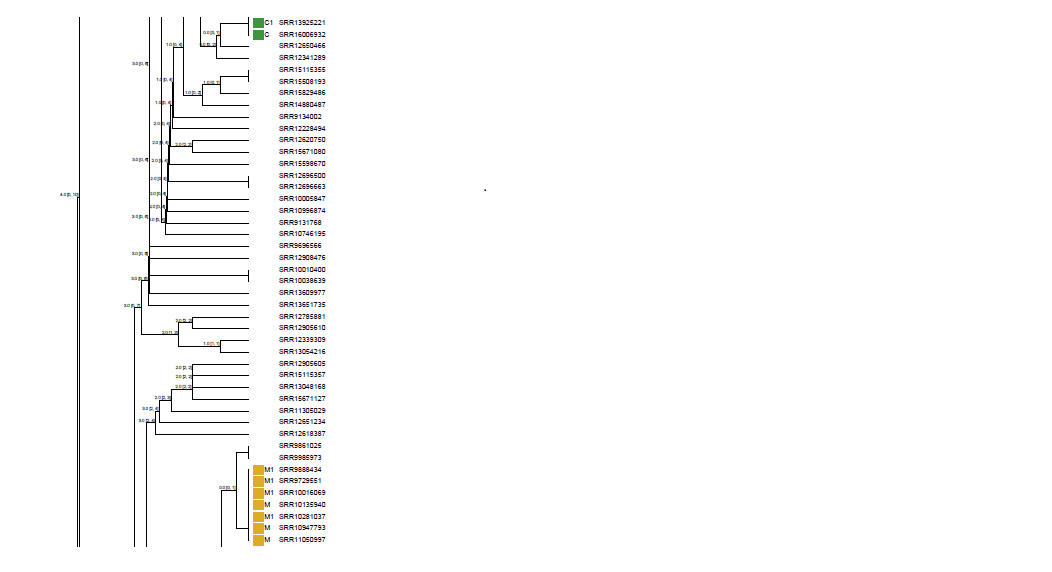


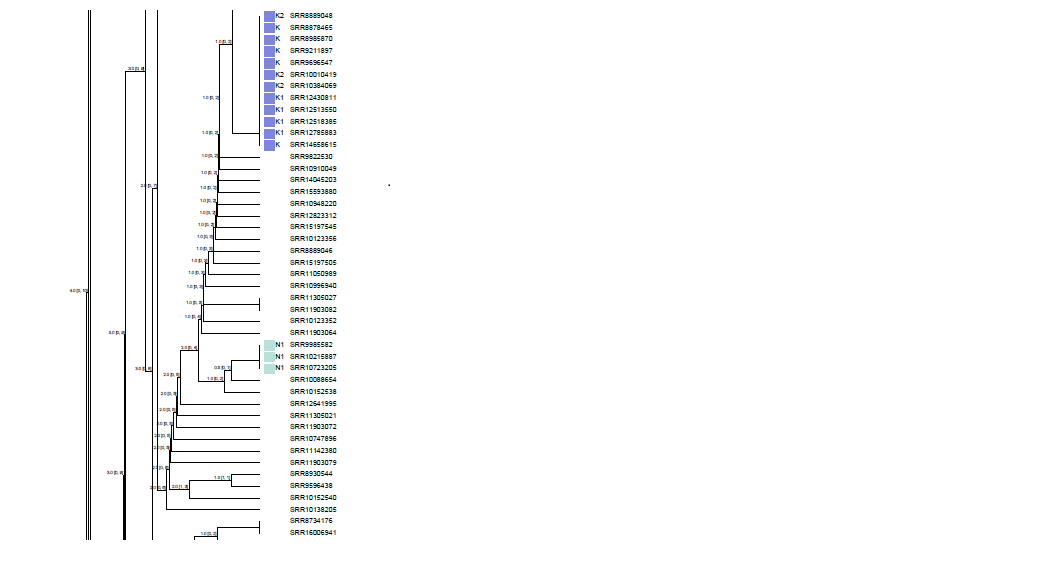


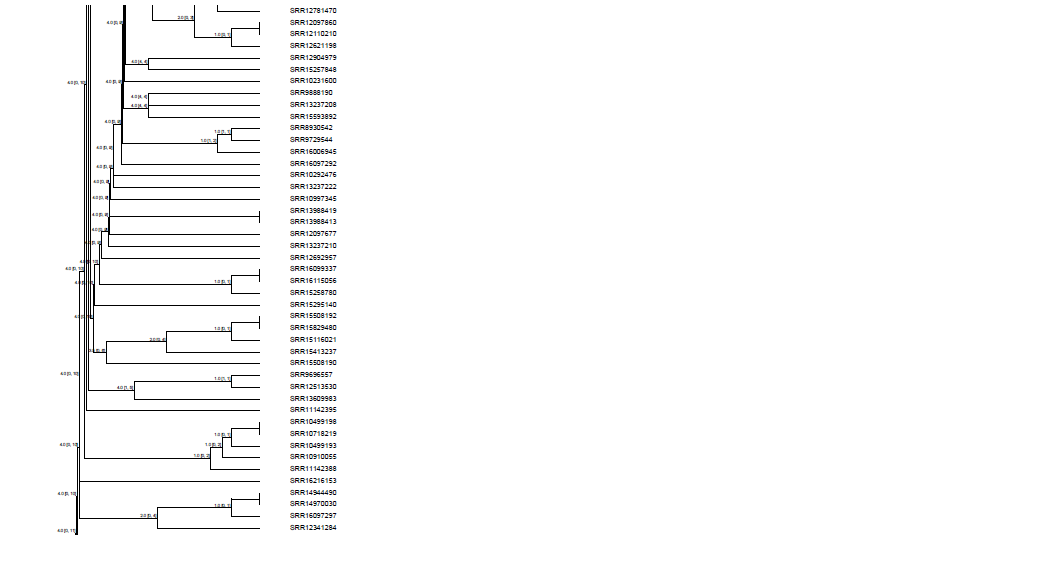


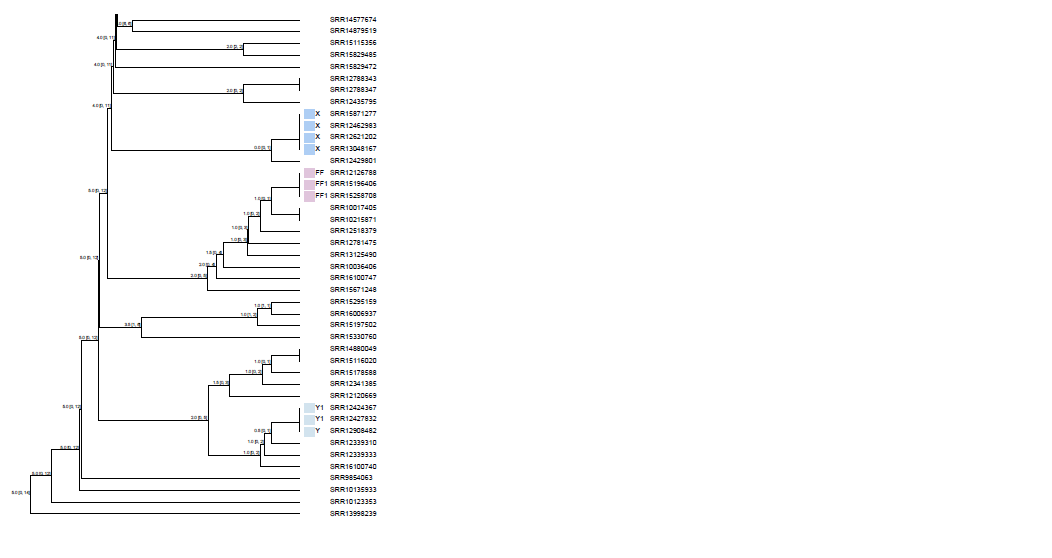

Supplement: Supplemental data [file Supp_FigS1.docx]
